# Synthesis, characterization, computational studies and biological activity evaluation of Cu, Fe, Co and Zn complexes with 2-butanone thiosemicarbazone and 1,10-phenanthroline ligands as anticancer and antibacterial agents

**DOI:** 10.17179/excli2017-984

**Published:** 2018-03-29

**Authors:** Tahmeena Khan, Iqbal Azad, Rumana Ahmad, Saman Raza, Shalini Dixit, Seema Joshi, Abdul Rahman Khan

**Affiliations:** 1 Department of Chemistry, Integral University, Dasauli, P.O. Bas-ha, Kursi Road, Lucknow-226026, UP, India; 2 Department of Chemistry, Isabella Thoburn College, 7, Faizabad Road, Lucknow-226007, UP, India; 3 Department of Biochemistry, Era's Lucknow Medical College and Hospital, Era University, Sarfarazganj, Hardoi Road, Lucknow-226003, UP, India; 4 Department of Analytical Chemistry, CSIR-Central Institute of Medicinal and Aromatic Plants, P.O.-CIMAP, Near Kukrail Picnic Spot, Lucknow-226015, UP, India

**Keywords:** thiosemicarbazone, mixed, 1,10-phenanthroline, docking, bioactivity

## Abstract

Mixed-ligand metal (II) (M=Cu, Fe, Co and Zn) complexes containing 2-butanone thiosemicarbazone and 1, 10-phenanthroline have been synthesized and characterized by melting point, FT-IR, ^1^H-NMR, UV-spectrophotometry and molar conductance measurements. All the complexes were soluble in DMSO and DMF. They were thermally stable with high melting points. The computational studies of the complexes were also performed to assess toxicity potential, bioactivity score prediction and drug likeliness assessment based on various drug filters. All the complexes showed no Veber's violations whereas only Cu complex showed one Lipinski's violation. Almost all synthesized compounds were predicted to have no toxic effects. Some of them showed positive bioactivity as enzyme inhibitors. Molecular docking of the complexes was also performed against ribonucleotide diphosphate reductase (RR) and topoisomerase II (Topo II) for minimum binding energy (kcal/mol) calculations. Cu complex was found to have minimum binding energy (-101.13 kcal/mol) released on interaction with Topo II showing a high affinity towards the enzyme, whereas Fe complex had the lowest binding energy (-99.8349 kcal/mol) when docked with RR. The results were compared with two standard drugs i.e. doxorubicin HCl and tetracycline. The ligand was tested for its potential anticancer activity against MDA-MB-231 cell line using MTT assay. Antibacterial activity of the complexes was tested against *Staphylococcus aureus* and *Escherichia coli* using the disc diffusion method. Cu (II) complex showed maximum activity against the MDA cells and also exhibited mild antibacterial activity against *S. aureus*.

## Introduction

Derivatives of 1,10-phenanthroline (1,10-phen) have been widely used in coordination chemistry (Chelucci et al., 2007[7]). Many of the derivatives have been shown to possess various biological activities (Liu et al., 2001[31]; Sammes and Yahioglu, 1994[37]). 1,10-phenanthroline has a rigid framework and possesses good chelating properties and can bind to many metal ions. The presence of two nitrogens enhances the coordination and charge transfer ability (Felder-Flesch et al., 2001[12]; Liu et al., 2005[30]). Thiosemicarbazones, an important class of Schiff based ligands possess good chelating properties. Transition metal complexes with thiosemicarbazone ligands have shown various pharmacological properties (Kovala-Demertzi et al., 2007[23], 2003[24]). Thiosemicarbazones containing heterocyclic donor atoms like sulphur and oxygen have shown an array of biological activities (Gupta and Narayana, 1997[15]; Khan et al., 2013[20]). The activity spectrum of the thiosemicarbazones can be enhanced as shown by their dependence on their substituents. Minor modifications in the thiosemicarbazones can lead to significant changes in biological activity. 1,10-phen has two aromatic nitrogens whose unshared electron pairs can bind to cations (Sammes and Yahioglu, 1994[37]) providing additional binding sites. The π-electron deficiency makes it a good π-acceptor (Farrell, 1989[11]). 

## Materials and Methods

Computational studies were performed using computer aided softwares. Molecular docking was done using iGEMDOCK version 2.0 and OSIRIS data warrior version 4.6.1 was used to assess toxicity whereas Molinspiration version 2016.03 was used to calculated bioactivity score and other physicochemical properties. The structures of receptors topoisomerase II (Topo II) (PDB i.d. 4GFH) and ribonucleoside diphosphate reductase (RR) (PDB i.d. 5CNT) were downloaded from protein data bank (http://www.rcsb.org/pdb/home/home.do); Biovia Discovery Studio visualizer version 16.1.0 and Lig Plus plot version 1.4.5 were used to visualize docking poses. All the chemical structures were drawn in Chem Draw Professionals version 15.1. The metabolic sites in the ligands were predicted using Metaprint 2D in Bioclipse version 2.6.2. All the chemicals used in the study were of A.R. grade and purchased from commercial suppliers and used without any further purification. Hydrated salts of metals viz. Cu(CH_3_COO)_2_.H_2_O, ZnSO_4_. 7H_2_O, CoCl_2_.6H_2_O and FeSO_4_.7H_2_O, were used for complex formation. IR spectra were recorded on a Bruker vertex 70 IR spectrophotometer in the frequency range 4000-500 cm−^1^ using KBr pellet method. ^1^H NMR spectra were recorded on a Bruker spectrophotometer on 500.1 MHz at 295 Kin the range of 0-10 δ using tetramethylsilane (TMS) as an internal standard and run in dimethyl sulfoxide (DMSO)-d_6_. Melting points were assessed in Ambassador electrical melting point apparatus up to 400 °C by the open glass capillary method. UV spectra were recorded on a SpectraMax-5 spectrophotometer (Molecular Devices) in the range 200-400 nm. Conductivity measurements were made on an EI Deluxe conductivity meter, Model-601, in DMSO (1.0 × 10^-^^3 ^mol). For biological activity evaluation, 0.4 % Trypan blue, PBS (pH = 7.2, 1×), 0.25 % trypsin-EDTA (1×), DMEM/F-12 (1×) (Dulbecco's modified Eagle's medium), and antibiotic/antimycotic solution (100×) were obtained from Gibco, Life Technologies, whereas fetal bovine serum (FBS) and MTT were obtained from HiMedia. DMSO was purchased from Calbiochem. MDAMB- 231 (human breast carcinoma, ER-, tumorigenic and invasive), abbreviated as MDA cells were obtained from the National Centre for Cell Science (NCCS), Pune, India. 

### Molecular docking studies

The molecular docking studies of the synthesized compounds were done using iGEMDOCK software. iGEMDOCK is a graphical interface which recognizes pharmacological interactions (Hsu et al., 2011[16]). Before docking, the ligands' energy minimization was performed by Merck Molecular Force Field (MMFF94). Docking studies help understand the molecular interaction with two protein targets, topoisomerase II (Topo II) and ribonucleotide diphosphate reductase (RR) to investigate the potential binding mode and energy. 

### Toxicity potential assessment

The complexes were computationally screened for any mutagenic, tumorigenic, irritant and reproductive toxicity risks*.* The toxicity risk was predicted by means of pre-computed set of structural fragments. The prediction of different properties of molecules in the early stage is a vital step in drug discovery and development process. Toxic parameters of the ligand and complexes were generated by OSIRIS Data Warrior software version 4.6.1.

### Bioactivity score prediction

Overall medicinal potential of a compound is predicted from its drug score. Through Molinspiration, the bioactivity score of the synthesized compounds against regular human receptors such as G-protein coupled receptors, nuclear receptor ligands, ion channel modulators, kinase inhibitors, nuclear receptors, proteases and enzyme inhibitors. As a general rule, greater is the bioactivity score, higher is the probability that investigated compound would be active. Therefore, a molecule having bioactivity score of more than 0.0 is most likely to possess considerable biological activities in clinical trial stage, while values ranging from -5.0 to 0.0 are expected to be moderately active and if the score is less than -5.0, it is presumed to be inactive (Verma, 2012[40]).

### Evaluation of drug likeliness

Drug likeliness of the complexes possessing acceptable physicochemical properties was done using the following filtering rules:

**1. Lipinski's rule of five:** Based on a set of counting rules, it is an important scale used for lead optimization. The rule describes molecular properties of a proposed compound explaining several pharmacokinetic parameters. A molecule is compatible with Lipinski's rule if:

1. its molecular weight is less than 500

2. the calculated logarithm of the octanol-water partition coefficient (cLogP) is less than 5

3. there are less than 5 hydrogen bond donor atoms

4. the sum of the number of nitrogen and oxygen atoms is less than 10.

The rule has achieved widespread acceptance while defining the limiting properties of most orally active drugs which are absorbed by passive mechanisms (Lipinski et al., 2001[29]).

**2. Veber Rules: **Veber et al. (2002[39]) observed that reduced molecular flexibility and low polar surface area as two important predictors of good oral bioavailability. Membrane permeability is an important requirement for oral bioavailability (Veber et al., 2002[39]). Reduced polar surface correlates with increased permeation rate than lipophilicity (cLogP) does and with the increase in number of rotatable bonds permeation decreases significantly. Following two criteria to be met by a potential drug candidate for oral bioavailability:

1. 10 or fewer rotatable bonds

2. Polar surface area equal to or less than 140 Å^2^ (or 12 or fewer H-bond donors and acceptors)

All the synthesized complexes showed no violations for Veber's filter. 

**3. Ghose filter: **Molecular lipophilicity and molar refractivity of drug molecules are important features which strongly influence receptor binding, cellular uptake and bioavailability. Being fragmental constants, they represent the hydrophobic and dispersive (van der Waals) interactions (Ghose and Crippen, 1987[13]). These properties may be used to develop a consensus definition of drug like character. The approach was used by Ghose and Crippen (1987[13]) to provide quantitative and qualitative characterization of known drugs under the comprehensive medicinal chemistry (CMC) database. The quantitative characterization was based on computed physicochemical properties such as logP, molar refractivity, MW and number of atoms. The qualifying range for different parameters as per Ghose filter is:

1. clogP should be between -0.4 and 5.6, with an average value of 2.52.

2. For MW, the qualifying range is between 160 and 480, with an average value of 357.

3. For molar refractivity, the qualifying range is 40-130, with an average value of 97.

4. For the total number of atoms, the qualifying range is between 20 and 70, with an average value of 48.

The effective range of these physicochemical properties can be used for designing drug-libraries and to test hypothetically proposed compounds before the stage of experimentation (Ghose et al.,1999[14]). 

**4. Leadlikeliness: **In their analysis to look for common sources of leads for drug discovery, Teague et al. (1999[38]) have described that leadlike molecules should have low affinity (>0.1 µM). Where the lead is an agonist the ED_50_ is taken to be a maximal value for the affinity of the molecule having the following requirements:

1. Molecular Weight <350

2. cLogP< 3.

### Metaprint 2D prediction

The probable metabolic sites in the primary ligand, 2-butanone thiosemicarbazone (L_1_) and secondary ligand 1,10-phenanthroline (L_2_) were predicted using Metaprint 2D (Lahari et al., 2015[27]). To understand the metabolic pathways of a new chemical entity, it is of utmost importance to know the metabolic mechanism. Metaprint 2D is a software predicting the probable sites most likely to undergo metabolism (Carlsson et al., 2010[5]). Through coloured circular fingerprints normalized occurrence ratio (NOR) is predicted. 

### Synthesis of the complexes

**Figure 1:**
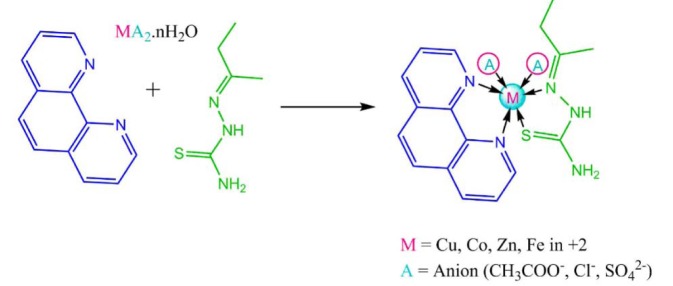
Reaction scheme of the synthesis

The ligand 2-butanone thiosemicarbazone (L_1_) was prepared using a previously reported procedure with some modifications (Kumar and Kumar; 2013[25], Khan et al., 2017[22]). The synthesis of complexes was done in 1:1:1 molar ratio. 0.003 M of the metal salt was dissolved in methanol with constant stirring on a magnetic stirrer. 0.003 M of ethanolic solution of 1,10phenethroline (L_2_) was added in the metal salt solution dropwise and the mixture was stirred for another half an hour. To the metal and secondary ligand solution, 0.003 M of ethanolic 2-butanone thiosemicarbazone was added and the contents were stirred for about 45 minutes. The contents were refluxed for 4-5 hours after which coloured precipitate was obtained which was filtered under suction and washed with ethanol and dried in desiccator. Same procedure was repeated every time. The synthetic scheme of the reaction is presented in Figure 1(Fig. 1).

### Biological activity evaluation

MDA-MB-231 cell line was maintained by sub culturing and passaging as monolayers 25- and 75-cm^2^ cell culture flasks (Nest, Tarsons) at 37 °C in a 5 % CO_2_ incubator at 95 % humidity for producing HCO_3_ buffering capacity (Khajah et al., 2013[19]) in Cell and Tissue Culture Lab, Dept. of Biochemistry, Era's Lucknow Medical College, Era University, Lucknow. The cells were maintained at pH 7.4 in DMEM containing phenol red as a pH indicator and supplemented with 5 % FBS. The medium, prior to being used in cell culture experiments, was vacuum filtered using a Corning filtration system (Corning®, Sigma-Aldrich).

### Methyl tetrazolium assay (MTT)

MTT assay was performed as per published protocol (Mosmann, 1983[34]). The experiment was performed in 96-well microtiter tissue culture plates (Linbro, MP Biomedicals). MDA cells were seeded at a density of 10^4^ in 200 μL of medium in a 96-well microtiter tissue culture plate and cultured in a humidified 5 % CO_2_ incubator at 37 °C for 24 h. 20-100 μM of complexes in 50 % DMSO were freshly prepared in culture media by serial dilution was carried out in cell culture media so that the final concentration of DMSO in the well did not exceed 0.5 % (v/v). Three control wells containing the medium only were also included as blank. After an incubation period of 24 h, cells were treated with the complexes for 48 h and the experiment was performed in triplicates. Equal volumes of 50 % DMSO (in cell culture media) were used as vehicle controls. 50 % DMSO (in cell culture media) in equal volumes were used as vehicle controls. At the end of treatment, cell culture medium containing varying amounts of complexes was removed and 20 μL of MTT (stock made in PSS at 5.0 mg/mL) reagent was added to each well and incubated for 4 h. Thereafter, MTT was removed and formazan crystals were dissolved in 200 μL of DMSO. The plates were read in a Bio-Rad PW41 ELISA plate reader at a wavelength of 570 nm with a reference wavelength of 630 nm. Percentage cell viability (Y-axis) was calculated from absorbance and plotted against concentration in micromolar (X-axis).

% Cell survival was calculated as = {(AT − AB)/(AC − AB)} × 100 

Where: 

AT = Absorbance of treatment well

AB = Absorbance of blank

Ac = Absorbance of control well

 % cell inhibition = 100 − Cell Survival.

### Antibacterial activity

The *in vitro* antibacterial activity of the complexes was evaluated against *S. aureus* (Gram-positive) and *E. coli* (Gram-negative) bacteria by the disc diffusion method (Al-Amiery et al., 2010[1]) using Mueller-Hinton agar (MHA) medium. The bacteria were subcultured in the agar medium and were incubated for 24 h at 37 °C. The discs (sterile filter paper discs, Whatman No. 1.0), having a diameter of 5 mm, were then soaked in the test solutions with the appropriate equivalent amounts of the ligand and its four complexes dissolved in sterile 50 % DMSO at concentrations of 2-30 mg/disc and placed on lawn culture of the respective microbial organism and stored in an incubator for the previously mentioned period of time. Formation of inhibition zone (if any) around each disc was measured, and the results were recorded in the form of inhibition zones as a function of diameter (mm). To clarify any effect of DMSO (used as a vehicle for the dissolution of the ligand and its complexes) on biological screening, 50 % DMSO was used as negative control where it showed no activity against any bacterial strains. Tetracycline was used as a positive control.

## Results and Discussion

### Molecular docking

**Figure 2:**
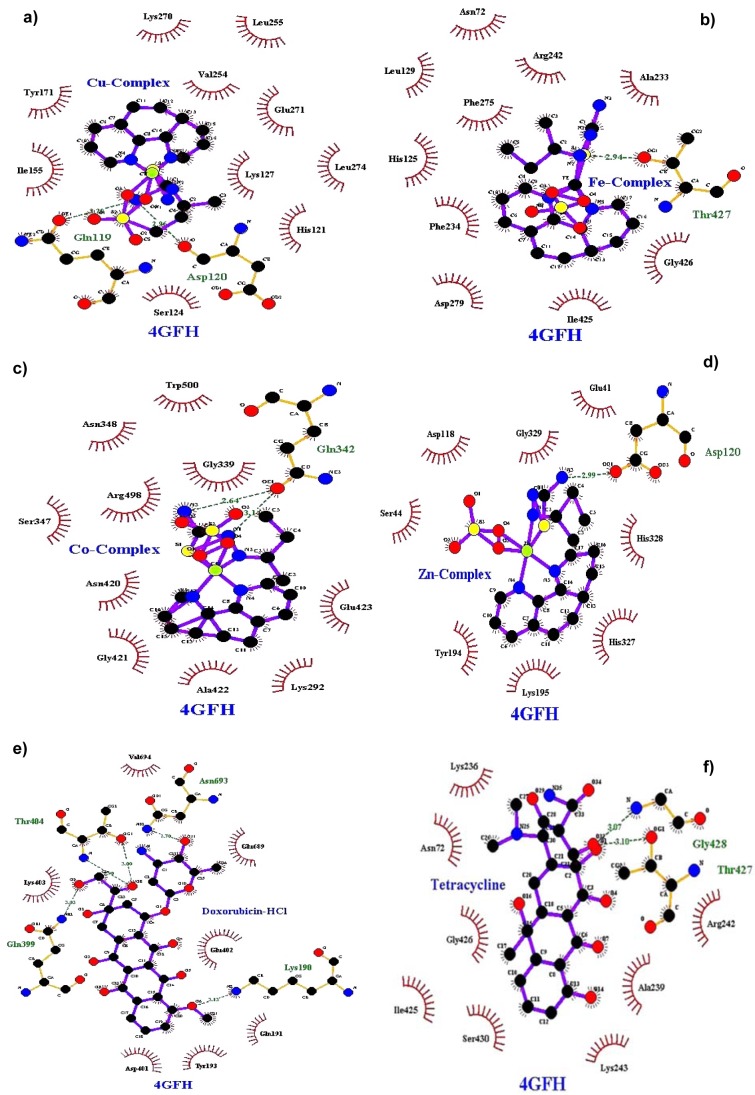
Docking poses of Topo II with a) Cu(II) complex, b) Fe(II) complex, c) Co(II) complex, d) Zn(II) complex, e) Doxorubicin HCl, f) Tetracycline

**Figure 3:**
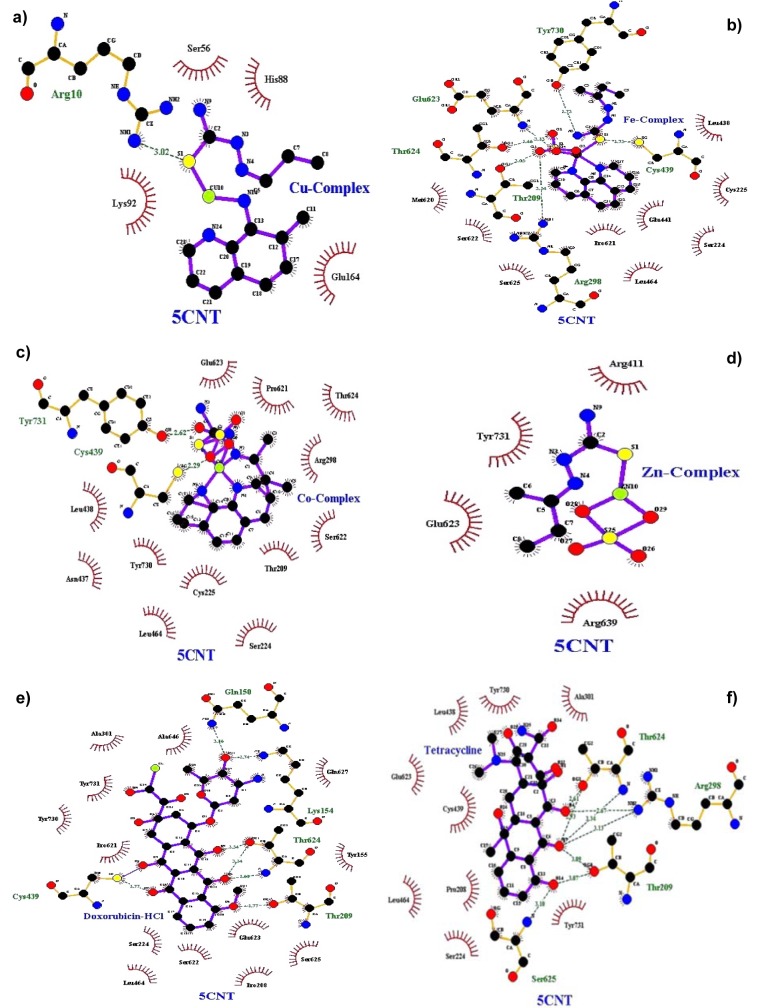
Docking poses of RR with a) Cu(II) complex, b) Fe(II) complex, c) Co(II) complex, d) Zn(II) complex, e) Doxorubicin-HCl, f) Tetracycline

Molecular docking studies allow us to characterize the behavior of molecules in the binding site of target proteins. Docking helps to understand and elucidate fundamental biochemical processes. The synthesized mixed-ligand metal complexes and reference drugs were subjected to molecular docking studies using the iGEMDOCK software to understand the drug molecule interaction with RR and Topo II enzymes in terms of minimum binding energy (kcal/mol), number of hydrogen bonds, pi-pi interactions, pi-sigma interactions and atomic charge interactions, the residual amino acids in proteins interacting with the complexes. Thiosemicarbazone compounds have been found to inhibit RR and Topo II (Khan et al., 2015[21]). iGEMDOCK is a useful software which provides associative interfaces to construct the binding sites of the target protein and the screening compound. Using the standard docking mode molecular docking was performed in a flexible manner. Genetic algorithm (GA) parameters were set as 200 population size and 70 generations in 10 numbers of solutions. After post-docking minimization, the pose with the minimal binding energy was used for accurate docking for greater accuracy. For accurate docking mode, GA parameters were set as 800 population size with 80 generations in 10 numbers of solutions. After docking is performed the software brings out protein interaction profile of van der Waals, electrostatic and hydrogen bonding interactions (Balavignesh and Srinivasan, 2013[4]). The target proteins RR and Topo II were downloaded in pdb format from protein data bank. The binding sites of the targets were prepared and the energy minimized compounds were imported. Docking process included preparation of molecules and then bonds, bond orders and flexible torsions were assigned to both the proteins and ligands. Docking was done between protein and ligand resulting in binding affinities in kcal/mol and docking run time. The compound giving the lowest binding energy is the best inhibitor (Middleton, 1998[32]). The overall binding interactions are given in Tables 1-2(Tab. 1)(Tab. 2) and docking poses with Topo II and RR are presented in Figures 2(Fig. 2) and 3(Fig. 3). 

The empirical scoring function of iGEMDOCK was estimated as

Fitness= vdW+Hbond+Elec.

vdW= vander Waal energy

Hbond= Hydrogen bonding

Elec= Electrostatic energy

The negative value of binding energy shows spontaneous binding process. Larger the negative value of the binding energy, greater is the ease of binding and greater is the possibility of the compound to be accepted as a drug molecule (Balavignesh and Srinivasan, 2013[4]). Cu complex was found to have minimum binding energy (-101.13 kcal/mol) released on interaction with Topo II showing a high affinity towards the enzyme, whereas Fe complex had the lowest binding energy (-99.8349 kcal/mol) when docked with RR. 

### Physicochemical parameters of the compounds

Table 3(Tab. 3) depicts molecular properties of the parent ligand and its proposed hetero-ligand complexes *versus* reference drugs by applying various drug rules and filters (vide supra). Almost all the synthesized complexes met with the criteria of the applied rules showing drug-like character. At the most one violation was obtained in each case. Molecular weights of three complexes were <500 and hence it could be predicted that these complexes can be easily transported, diffused and absorbed. Number of rotatable bonds in all the complexes were <10 indicating their lesser molecular flexibility. TPSA which is correlated with the hydrogen bonding of a molecule is a good indicator of the bioavailability of the drug molecule. Osiris tool identified the property based on summation of surface contributors of polar fragments (Ertl et al., 2000[10]). TPSA of all the synthesized complexes was in the range 48.83-101.4 that was well below the limit of <160 Å. 

### Toxicity potential

Table 4(Tab. 4) represents the toxicity potential assessment data of the complexes as calculated by OSIRIS Property Explorer. The software predicts the toxicity potential based on the similarities of the investigated compounds with the known compounds in its database. The computational toxicity risk assessment is necessary to avoid unsuitable or further drug screening, if the compounds are predicted to have adverse side effects on biological system (Balakrishnan et al., 2015[3]). The predictions are colour coded. Colour red indicates high risk of undesired effects while a green color predicts conformation to drug-like behavior and compatibility (Husain et al., 2016[17]). 

### Bioactivity score prediction

The bioactivity scores of all the proposed complexes are depicted in Table 5(Tab. 5). As a general rule, greater is the bioactivity score, higher is the probability that investigated compound would be active. Therefore, a molecule having bioactivity score of more than 0.0 is most likely to possess considerable biological activities, while values ranging from -5.0 to 0.0 are expected to be moderately active and if the score is less than -5.0, it is presumed to be inactive (Verma, 2012[40]). Against ICM and EI all the complexes showed good bioactivity score. Against GPCR and PI, Zn and Fe complexes showed positive bioactivity score. 

### Evaluation of drug likeliness

The compounds were evaluated on Lipinski, Veber, Ghose and Lead likeliness parameters as summarized in Table 6(Tab. 6). 

All the synthesized complexes showed no Ghose violation whereas the standard anticancer drug doxorubicin HCl showed two violations for Ghose filter. Likewise for Lipinski's filter only Cu(II) complex showed one violation and rest of the compounds had zero violations. Doxorubicin-HCl and tetracycline showed three and one violation respectively in case of Lipinski's filter. 

### Meta print 2D prediction for the ligands

**Figure 4:**
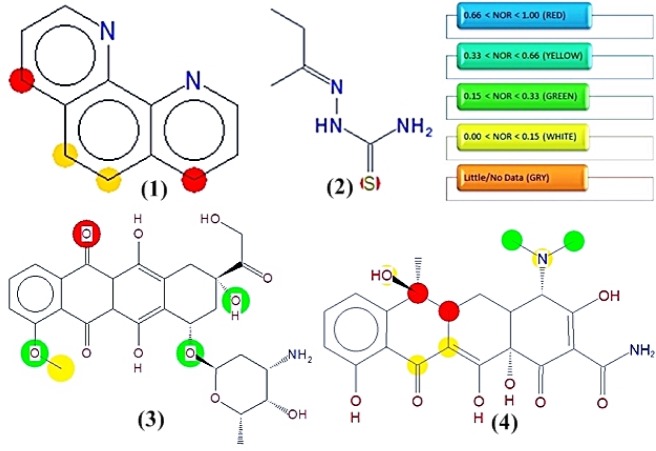
Predicted phase I metabolic sites in 1,10-phenanthroline and 2-butanone thiosemicarbazone *versus* reference drugs. Sites of metabolism are indicated by different colors viz. red color atoms will be most metabolized according to the probability of a metabolic site; Red: High, Orange: Medium, Green: Low, White: Very low and Grey: No data. 1,10-phenanthroline (1), 2-butanone thiosemicarbazone ligand (2), doxorubicin HCl (3), tetracycline (4)

While understanding the metabolic pathways of a new chemical entity (NCE) it is of great importance to understand how a compound would be metabolized in human beings. Metaprint 2D predicts the sites likely to undergo metabolism (Carlsson et al., 2010[5]) as shown in Figure 4(Fig. 4). The predictor uses circular fingerprints to indicate the sites of metabolism. The colour highlighting of an atom indicates its normalized occurrence ratio (NOR). A high NOR indicates a more frequently reported site of metabolism in the metabolite database. Different colours indicate different extents of metabolic sites. 1,10-phenanthroline was predicted to have several probable sites likely to undergo metabolism. 

### Characterization data

**2-butanone thiosemicarbazone- C_5_H_11_N_3_S**. The characterization data of the ligand has already been published in our previous manuscript (Khan et al., 2017[22]). Colour-Creamish white, Solubility: ethanol, DMSO, DMF Yield 90 %; MW-145; MP (°C) 99.

**1. [Cu (1,10-phen)(C_5_H_11_N_3_S) (CH_3_COO)_2_]- **Colour- Dark green, Solubility- DMSO, DMF, Yield 68 %, MW- 507.07, MP (°C) > 350, FTIR (KBr) (cm−^1^) 1581 (C=N Azomethine), 1518 C=N (Phen), 1426 (C=C), 774, 1059 (C=S) 3000-3302 (NH, NH_2_), 488 (M-N), λ_max_ (nm) 302, Molar conductance 12.4 Ω^−1^ cm^−1^ mol^−1^

**2. [Fe** **(1,10-phen)(C_5_H_11_N_3_S) SO_4_]- **Colour- Reddish-brown, Solubility- DMSO, DMF, Yield 75 %, MW- 477.34 MP (°C) >350, FTIR (KBr) (cm−^1^) 1589 (C=N Azomethine), 1513 C=N (Phen), 1425 (C=C), 726, 1055 (C=S) 3295 (NH) , λ_max_ (nm) 290, Molar conductance 24.0 Ω^−1^ cm^−1^ mol^−1^

**3. [Co (1,10-phen)(C_5_H_11_N_3_S)Cl_2_] - **Colour- Red, Solubility- DMSO, DMF, Yield 77 %, MW- 455.27, MP (°C) > 350, FTIR (KBr) (cm−^1^) 1579 (C=N Azomethine), 1514 C=N (Phen), 1413 (C=C), 726, 1101 (C=S), 3412, 3045, 2993 (NH, NH_2_), 420 (M-N), λ_max_ (nm), 301, Molar conductance 40.4 Ω^−1^ cm^−1^ mol^−1^

**4. [Zn** **(1,10-phen)(C_5_H_11_N_3_S) SO_4_]- **Colour- Creamish white, solubility- DMSO, DMF, Yield 78 %, MW- 486. 87, MP (°C) > 350, FTIR (KBr) (cm−^1^) 1583 (C=N Azomethine), 1518, C=N (Phen), 1427 (C=C), 725, 1037 (C=S) 3200 (NH), 425 (M-N), λ_max _(nm) 297, Molar conductance 8.9 Ω^−1^ cm^−1^ mol^−1^.

The spectral analyses revealed the formation of complexes. All the complexes were soluble in DMSO and DMF and obtained as coloured solids with high melting points. The IR spectra predicted the bonding sites of both primary and secondary ligand. The IR frequencies showed that 2-butanone thiosemicarbazone coordinates with the metal centre through the azomethene nitrogen (Deepa and Aravindakshan, 2000[9]; Joseph et al., 2006[18]) and the thione sulfur (Chandra et al., 2001[6]; Youssef et al., 2005[43]), because the C=N and C=S frequencies decrease in case of metal complex formation. The IR spectrum of the free 1,10-phen ligand shows a strong band at around 1570 cm^-1^ due to the stretching frequency of C=N present in the moiety. In complexes the band was shifted to lower frequencies indicating that the two nitrogens coordinate to the M(II) ion in complexes (Aljahdali and El-Sherif, 2013[2]). The negative shift of the C=S band in the complexes confirm the coordination via the thione sulfur (West et al., 1995[42], 1993[41]; Kurup and Joseph, 2003[26]). Presence of new bands between 400-500 cm^-1^ in the IR spectra of complexes indicates the M-N bond (Raman et al., 2009[35]). As compared with free 1,10 phenanthroline, the C=C stretching band (1438 cm^-1^) shifted to lower frequencies in complexes due to coordination (Costa Pessoa et al., 1999[8]). High melting points of the complexes were another indication of coordination. The ^1^H NMR spectra of the complexes were recorded in DMSO-d_6_. With respect to free ligand the chemical shift values were shifted in the complexes confirming the complex formation. The multiplet due to 1,10-phen aromatic protons was obtained between 7.5-8.9 ppm. The signal obtained at 8.9-9.9 ppm was due to NH proton whereas the NH_2_ peak was obtained around 9.0 ppm. The signals due to CH_3_, CH_2_ groups in 2-butanone thiosemicarbazone ligand were obtained upfield between 1.00-2.8 ppm. The UV-spectrophotometry data showed the shifting of λ_max_ at 267 nm in free ligand (π-π*) transition to higher values corresponding to n-π* transition (Red shift) indicating the ligand to metal charge transfer (LCMT). Shifting of the UV peaks to higher wavelength shows Bathochromic shift or red shift which clearly indicates the complex formation (Leovac et al., 2007[28]). Molar conductance values can help in predicting whether the counter ion satisfies the secondary valency. Conductance depends upon the ionization behaviour of the complex as larger number of ionizable ions would produce greater molar conductance (Refat, 2007[36]). All the four complexes had very low conductance values which were an indicative of the non-ionizing nature of the complex (Mohamed et al., 2009[33]). These results are consistent with the light-electrolytes (1:1) nature of the complexes. These data indicate that the ligands have coordinated with the metal ions. 

### MTT assay results

**Figure 5:**
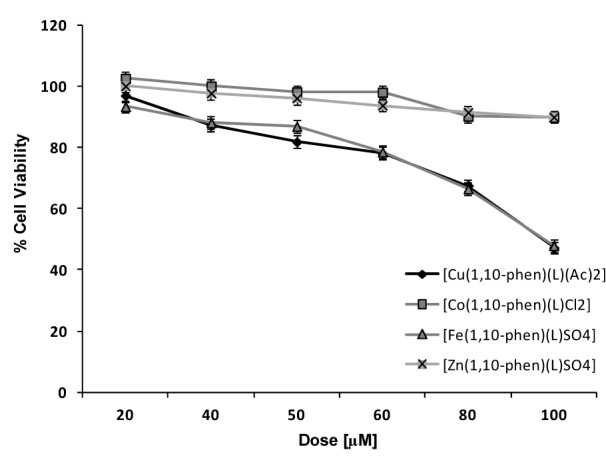
Dose response curves of effect of Cu, Co, Fe and Zn complexes on viability of MDA cells *in vitro* using MTT assay. Final concentration of DMSO in each well did not exceed 0.5 % (v/v). Results were expressed as mean ± SD of experiments done in triplicates.

Figure 5(Fig. 5) depicts the dose-dependent effect of the Cu, Co, Fe and Zn complexes on MDA using the MTT assay. Cu(II) complex was most effective with approximately 49 % cell viability followed by Fe(II) complex having 57 % cell viability approximately. The IC_50 _of the complexes was in between 80-100 µM range. 

### Antibacterial activity

**Figure 6:**
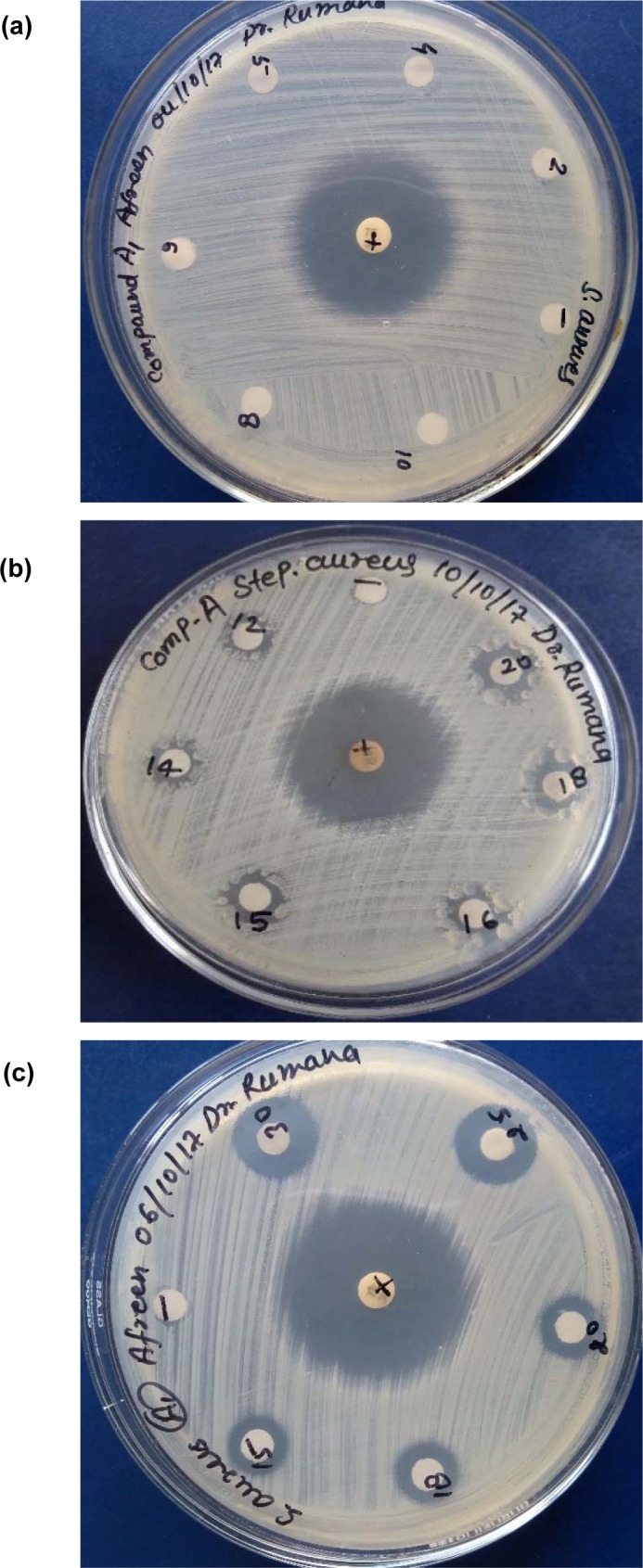
Figure 6 (a-c): Antibacterial activity of Cu(II) complex against *S. aureus* in different concentration ranges (mg/mL) (a) 2-10, (b) 12-20, 15-30

The complexes were also evaluated for their potential antibacterial activity against *S. aureus* and *E. coli* in a concentration range between 2-30 mg/mL. The Fe, Co and Zn complexes did not exhibit any significant activity against both species. However, Cu complex exhibited appreciable antibacterial activity against *S. aureus* (Figure 6(Fig. 6), Tables 7-8(Tab. 7)(Tab. 8)).

## Conclusion

Four mixed ligand complexes containing 2-butanone thiosemicarbazone and 1,10-phenanthroline were synthesized and characterized by various spectroscopic techniques. The complexes were evaluated for their anticancer potential against MDA cell line and for their antibacterial activity against *E. coli* and *S. aureus*. Octahedral geometry was assigned to the complexes. Through spectroscopic studies it was concluded that both ligands coordinated to the metal ion. The complexes possessed appreciable thermal stability. Computational studies are a helpful tool to predict overall activity spectrum of a synthesized molecule. Understanding various aspects of how a compound affects biological structures, various metabolic processes and pathways and overall impact on physiological response is of prime importance. The present study used different prediction rules to predict oral bioavailability of compounds which can lead to a path of the discovery of new safer drugs. In the study computer-aided prediction of physicochemical properties, toxicity potential and bioactivity score was done. The synthesized compounds possessed positive bioactivity scores against some of the human receptors like ion channel modulator and enzyme inhibitors. OSIRIS Property Explorer version 4.5.1 was used to predict toxicity and druglikeness showing appreciable results. Molecular docking was done with two target proteins to know binding interactions in terms of van der Waals, H-bonding and electrostatic forces of attraction. The Cu(II) complexes exhibited maximum anticancer and antibacterial potential. In future, these complexes would be tested on other cancer cell lines along with their antioxidant and DNA cleavage activities. For validation of toxicity and other side effects of the complexes, they need to be tested on normal cell lines as well, in future. 

## Acknowledgements

The authors gratefully acknowledge Dr. E.S. Charles, President, Isabella Thoburn College, Lucknow, for providing necessary facilities to carry out the lab work. The authors are also thankful to Mr. Mohsin Ali Khan, Secretary, EET and Chairman Research, Era's Lucknow Medical College and Hospital, Era University, Lucknow, for extending the necessary facilities and co-operation for carrying out biological activity evaluation studies. The authors are also thankful to the RandD office of Integral University, Lucknow, for editing and communication number (IU/RandD/2017-MCN000202) for the manuscript.

## Conflict of interest

The authors declare that they have no competing interests.

## Figures and Tables

**Table 1:**
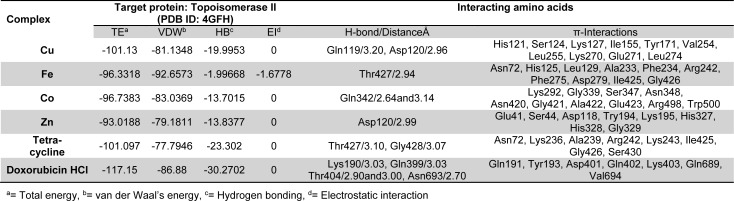
Docking results of synthesized compounds against Topo II

**Table 2:**
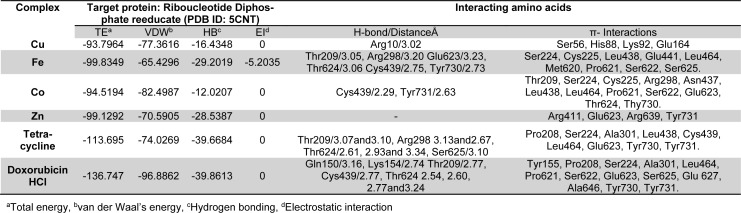
Docking results of synthesized compounds against RR

**Table 3:**
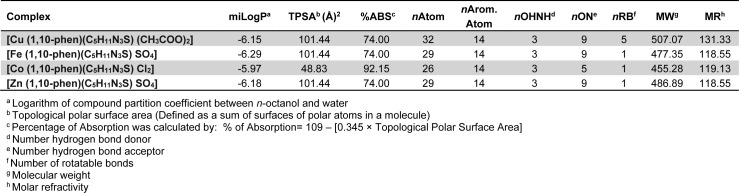
Physicochemical parameters of the compounds

**Table 4:**
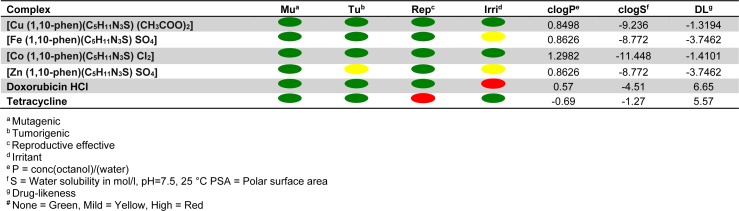
Drug-likeness and toxicity calculations of metal complexes based on Osiris property explorer

**Table 5:**
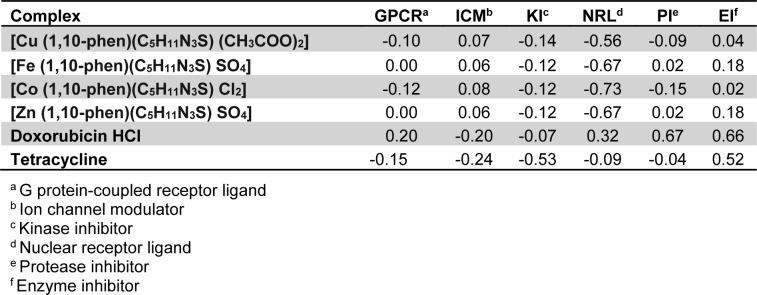
Bioactivity score of the compounds

**Table 6:**
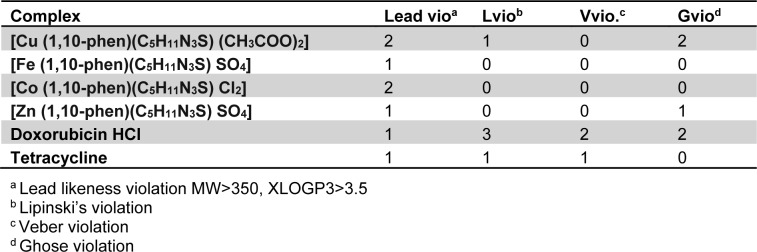
Drug-likeliness parameters

**Table 7:**
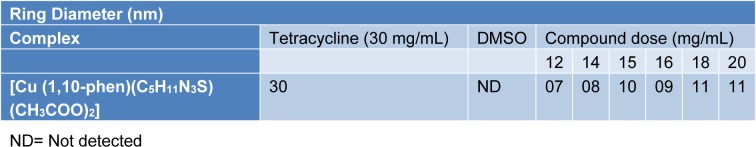
Comparison of MIC values (in mg/mL) of Cu(II) complex and standard antibiotic tetracycline against *S. aureus*

**Table 8:**
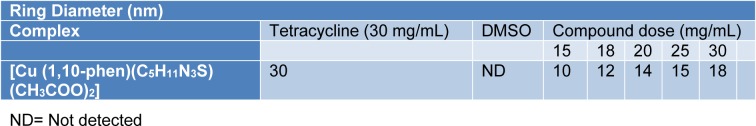
Comparison of MIC values (in mg/mL) of Cu (II) complex and standard antibiotic tetracycline against *S. aureus*
